# The Role of the Genital Tract Microbiome in Human Fertility: A Literature Review

**DOI:** 10.3390/jcm14092923

**Published:** 2025-04-24

**Authors:** Giuseppe Gullo, Marini’ Satullo, Valentina Billone, Lina De Paola, Stamatios Petousis, Yuliia Kotlik, Chrysoula Margioula-Siarkou, Antonio Perino, Gaspare Cucinella

**Affiliations:** 1 Department of Obstetrics and Gynaecology, AOOR Villa Sofia—Cervello, University of Palermo, 90127 Palermo, Italy; marinisat@gmail.com (M.S.); valentina.billone@gmail.com (V.B.); yuliia.kotlik@gmail.com (Y.K.); antonio.perino@unipa.it (A.P.); gasparecucinella1@gmail.com (G.C.); 2 Department of Anatomical, Histological, Forensic and Orthopedic Sciences, Sapienza University of Rome, 00161 Rome, Italy; lina.depaola@uniroma1.it; 3 2nd Department of Obstetrics and Gynaecology, Aristotle University of Thessaloniki, 541 24 Thessaloniki, Greece; petousisstamatios@gmail.com (S.P.); m.s.chrysoula@gmail.gr (C.M.-S.)

**Keywords:** microbiome, maternal health, human fertility, infertility, assisted reproductive technology

## Abstract

**Background/Objectives**: Infertility is a multifactorial condition influenced by various factors, including dysbiosis and alterations in the genital tract microbiome. Recent studies emphasize the microbiome’s significant role in influencing a woman’s fertility potential, thereby affecting the chances of spontaneous conception and the outcomes of assisted reproductive treatments. Understanding the microbial characteristics and unique features of a healthy genital microbiome, as well as how changes in its composition can impact fertility, would allow for a more comprehensive and personalized approach to managing assisted reproductive treatments. The microbiome also influences pregnancy outcomes, and restoring its balance has been shown to improve fertility in infertile couples. The human microbiome plays a key role in maintaining the body’s overall health. Disruptions in microbiome balance among women of reproductive age can contribute to a range of pregnancy-related complications, with notable consequences for both maternal and fetal well-being. Emerging research has highlighted a connection between the reproductive tract microbiome and outcomes of assisted reproductive technologies (ART), suggesting that re-establishing a healthy microbial environment may enhance fertility in couples facing infertility. **Methods**: We conducted a search on PubMed using the keywords “microbiome”, “infertility”, and “ART” over the past 10 years. This article aims to provide an updated overview of the role of the microbiome in female reproductive health, with a focus on its implications for fertility treatment. **Results**: The microbiome has a significant role in influencing women’s fertility. **Conclusions**: Understanding the microbiome’s impact on fertility and pregnancy outcomes may lead to more effective and personalized approaches in fertility treatments, improving the chances of successful conception and pregnancy.

## 1. Introduction

The human body is colonized by billions of microbial agents, the majority of which are located in the gastrointestinal system and play a crucial role in human immunity. Disruption of this microbial flora can lead to what is known as microbial dysbiosis, a phenomenon that can be a risk factor for various diseases [1].

There is also the urogenital microbiota, which represents the active microbiota in female health. Indeed, urogenital dysbiosis often leads to the onset of conditions with a range of symptoms that can be particularly bothersome for the affected patient. Dysbiosis of both the female and male microbiota can also influence fertility [2].

In recent years, reproductive health, particularly in women and couples, has garnered greater attention in the scientific community. There is an increasing demand for reproductive care and the use of medically assisted procreation techniques. Beyond the specific individualized treatment offered to couples, more scientific research has focused on the impact that lifestyle, diet, infections, diseases, and microbial flora can have on fertility [3].

Infertility issues among couples have been rising in recent decades, and the WHO (World Health Organization) now considers infertility a medical condition, defining it as the absence of conception after 12 to 24 months of regular, unprotected intercourse [4]. Infertility affects about 10–12% of couples worldwide. This condition can affect the man, the woman, or both (couple infertility). However, it may also occur that a particular union between individuals is unable to conceive a life.

The microbiome is regarded as the dynamic set of all pathogenic, commensal, and symbiotic microorganisms in a specific area of the human body.

Microorganisms, along with their genetic information and the environment in which they interact, are collectively referred to as “the microbiome” [5].

Previous studies have shown that microbial homeostasis can help maintain health and well-being, just as dysbiosis can contribute to the development of disease states. In particular, the genital tract microbiome may play a crucial role in a woman’s fertility status, embryo implantation, and the maintenance of pregnancy [6,7,8,9].

In recent decades, the rise in infertility has led to the growth of medically assisted procreation techniques, providing hope to many couples facing reproductive challenges. Recently, numerous studies have compared the characteristics and composition of the genital microbiome with the outcomes of assisted reproductive technologies (ART) [10].

Gaining a detailed insight into the influence of the microbiome on assisted reproductive technologies (ART) remains an essential objective in the field. The purpose of our paper is to review, analyze, and compare the numerous studies and previous works that have examined the link between the microbiome and infertility and to explore the impact that the composition of the genital tract microbiota may have on the outcomes of IVF treatments.

## 2. Materials and Methods

**Figure 1:**
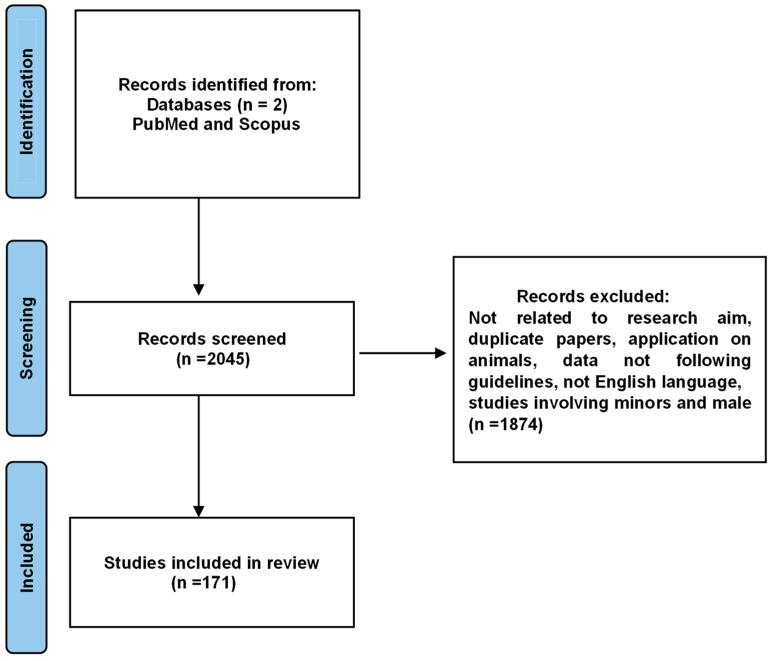
Prisma flow chart.

We performed a narrative review of the available scientific literature using the major databases PubMed and Scopus, focusing on studies published within the last 10 years. The keywords used included “microbiome infertility”, “microbiome fertility”, and “microbiome IVF”. After applying these search terms and narrowing the results to publications from the past decade, 2045 papers were identified. Subsequent filtering by the criteria “English language articles”, “human species”, “female sex”, and “adult (19+ years)” yielded 171 articles. Data from these studies were collected, and a manual search of the references within the selected articles was also performed (Figure 1).

The selected articles were reviewed, and from this pool, we chose the most relevant studies to our objective. We included references from papers that provided a more in-depth analysis of the characteristics of a healthy microbiome in both the upper and lower genital tract, as well as those that examined how changes in this microenvironment can lead to dysbiosis. We also placed particular emphasis on studies exploring the potential impact of genital microbiota alterations on IVF success rates and pregnancy outcomes. The purpose of this study was to review, analyze, and compare the existing literature on the relationship between the microbiome and infertility, with particular focus on how the composition of the genital tract microbiota may influence the outcomes of IVF treatments.

## 3. Results

### 3.1. Composition and Characteristics of the Genital Microbiome in Women of Childbearing Age

The vaginal microbiome is the collection of microorganisms that inhabit the human vagina. Like all microbiota, it includes bacteria, fungi, viruses, archaea, and protists, although the composition of microorganisms other than bacteria is not yet fully mapped [1,11]. Unlike the gut microbiome, which is influenced by lifestyle and diet, the vaginal microbiome is more strongly influenced by factors such as hormone secretion, sexual activity, hygiene practices, medications, sleep, genetics, ethnicity, and age [10,11].

In a healthy female vagina, *Lactobacillus* spp. predominantly colonize the area, starting from puberty. Specifically, a healthy vaginal microbiome is characterized by an abundance of *Lactobacillus* spp. in combination with low microbial diversity, which is associated with better reproductive outcomes. In contrast, an environment dominated by anaerobic species (e.g., *Gardnerella*, *Prevotella*, and *Atopobium*) may indicate dysbiosis. Vaginal dysbiosis is a significant risk factor for early pregnancy loss in patients undergoing assisted reproductive techniques (ART) [6,11,12,13,14,15].

Hormonal fluctuations during the phases of the menstrual cycle play a key role in shaping the microbiome. For example, estrogen levels increase during the follicular phase and reach their peak at ovulation. This rise in estrogen promotes the formation of glycogen deposits in the vaginal epithelium, thereby facilitating the expansion of *Lactobacillus* spp. in the vagina [16]. During pregnancy, the abundance of Lactobacillus species increases, resulting in a decrease in vaginal microbial diversity. These bacteria produce lactic acid [17], which contributes to a lower vaginal pH, creating an environment that inhibits the growth of pathogenic and less beneficial bacterial species [18]. Research has shown that pregnant women with higher vaginal microbial diversity face an increased risk of miscarriage and preterm birth [19]. Conversely, those with a Lactobacillus-dominated microbiota tend to experience fewer infections and reduced levels of inflammation [20].

Regarding the cervical tract, research has demonstrated the dominance of Lactobacillus (e.g., *L. crispatus* and *L. iners*) in 12 out of 13 cervical samples from healthy women and a strong correlation in the microbiome composition between vaginal, cervical, and endometrial samples from the same individual [21,22].

These findings, along with others, suggest that the microbiome undergoes progressive changes from the lower genital tract (LGT) to the upper genital tract (UGT), with a lower proportion of *Lactobacillus* spp. in the UGT compared to the LGT. In 2017, a research group demonstrated that cervical mucus samples showed a dominance of *Lactobacillus* spp. in the cervix [21,22,23].

Pelzer et al. demonstrated that *Lactobacillus* spp. was the most prevalent genus in endocervical samples, followed by *Gardnerella* spp., *Veillonella* spp., *Prevotella* spp., *Sneathia* spp., and *Fusobacterium* spp. [21,24].

Another study examining cervical samples reported *Acinetobacter* as the dominant genus (49%), followed by *Pseudomonas*, *Cloacibacterium*, and *Lactobacillus* [21,25].

In the past, before the introduction of next-generation sequencing, the uterine cavity was thought to be aseptic. Nowadays, it has become possible to describe microbial communities within the uterine cavity, though their origin remains unclear. It is hypothesized that uterine colonization may occur via the gut, oral cavity, bloodstream, and vaginal upwelling [26,27]. Extensive studies comparing differences and characteristics of the microbial flora of the lower genital tract and the uterine cavity have shown a more sparsely populated but more diverse bacterial flora in the upper genital tract [23].

The characteristics of the genital microbial flora of healthy women have been explored and described in several papers, where a preponderance of lactobacilli in a healthy microbiome is confirmed. In a research study, the microbiome of 58 women who had undergone hysterectomy for non-oncological reasons was analyzed using endometrial swabs; it was found that 95% of the patients had an abundant percentage in the UGT microbiome of the following bacteria: *L. iners*, *Prevotella* spp., and *L. crispatus* [27,28].

Endometrial fluid samples from 13 fertile women were examined; again, the dominance of lactobacilli was reconfirmed. Consequently, it is possible to describe two types of bacterial compositions at the endometrial level: Lactobacillus-dominant (LD), with lactobacilli accounting for at least 90% of the microbiome, or non-Lactobacillus-dominant (NLD), with lactobacilli present for less than 90% of the flora. Other microorganisms often found in endometrial fluid samples are Bifidobacterium, Gardnerella, Prevotella, and Streptococcus [27,29].

In general, more than 20 species of *Lactobacillus* have been identified in the genital tract environment. *Lactobacillus* spp. account for 90–95% of the total bacterial population in the reproductive tract, with four numerically dominant species: *Lactobacillus crispatus*, *Lactobacillus iners*, *Lactobacillus jensenii*, and *Lactobacillus gasseri* [30,31,32,33].

*Lactobacilli* in the female genital tract help maintain a favorable environment for embryo implantation through the production of lactic acid, hydrogen peroxide, bacteriocins, hydroxyl radicals, and probiotics [30,31,32,33].

Another study pointed out that Lactobacillus was the preponderant genus in both endometrial tissue and endometrial fluid, while other bacteria, e.g., Anaerococcus, Atopobium, Bifidobacterium, and Gardnerella, were present in smaller amounts [27,34].

### 3.2. Impact of the Genital Tract Microbiome on Infertility

Bacterial vaginosis is one of the most common gynecological pathological conditions and is among the leading reasons for seeking gynecological examinations in developed countries. It is characterized by an altered microbial flora, resulting in an increase in Gardnerella vaginalis at the expense of Lactobacilli. Bacterial vaginosis is associated with conditions such as endometritis, infertility, and pelvic inflammatory disease [8]. Despite being referred to as “bacterial vaginosis”, this condition affects both the lower and upper genital tracts [30,35].

The microbiome of patients undergoing IVF cycles has been studied, and it was found that approximately 40 percent of patients undergoing medically assisted reproduction techniques exhibited altered vaginal microbial flora [30,36,37].

In 1955, Gardner and Dukes identified *Gardnerella vaginalis* as one of the primary microorganisms responsible for bacterial vaginosis, initially naming it *Haemophilus vaginalis*. Bacterial vaginosis is a polymicrobial disease caused not only by *Gardnerella vaginalis* but also by other anaerobic bacteria that produce biofilms. Early diagnosis of this condition is crucial to prevent significant sequelae. The Amsel criteria, published in 1983, are still widely used for diagnosing bacterial vaginosis [30,38,39,40,41]. To diagnose bacterial vaginosis using the Amsel criteria, at least three of the following four conditions must be met:

1. Homogeneous, thin, grayish-white vaginal discharge: The discharge should be thin and uniform in appearance, often with an off-white or grayish color.
2. pH of the vaginal discharge > 4.5: Normal vaginal pH is typically between 3.8 and 4.5, and a pH greater than 4.5 is indicative of bacterial vaginosis.
3. Positive “whiff” test: A strong, fishy odor is released when a small amount of vaginal discharge is mixed with 10% potassium hydroxide (KOH). This odor is due to the production of amines by the anaerobic bacteria associated with BV.
4. Clue cells on wet mount: Clue cells are vaginal epithelial cells that are covered with bacteria, making the borders of the cells appear fuzzy or “clue-like” under a microscope [40,41].

Other studies have determined that the essential factors for the presence of BV are a lack of H2O2-producing lactobacilli associated with overgrowth of *Gardnerella vaginalis* and other anaerobic Gram-negative bacteria and anaerobic Gram-positive cocci [42].

Beyond the individual microbial species that populate the genital microbiome, the relative abundance of the various species is crucial.

In the past, Gardner and Duke demonstrated that a reduction in the percentage of lactobacilli, in favor of an increase in the relative abundance of *Gardnerella* and other anaerobes, promotes the occurrence of disease states.

More recently, 16S rRNA sequencing of isolates from BV patients has revealed extraordinary diversity among the species in the patients’ flora. This has led to the understanding that it is not a single pathogen responsible for the disease state but rather that BV is defined as an “ecological disorder of the vaginal microbiome” [43].

The biofilm of BV primarily includes *Gardnerella vaginalis* and *Atopobium vaginae*, with lower concentrations of lactobacilli [44,45].

Neither the immune system nor antibiotics can eradicate all pathogenic microorganisms responsible for BV, meaning that such infectious disease states can become persistent and have a high probability of recurrence. This can negatively impact efforts to achieve pregnancy and lead to increased reliance on IVF techniques [45,46].

### 3.3. Impact of Genital Tract Microbiome on Assisted Reproductive Technology

There has been considerable research, particularly in recent years, exploring the correlations between the microbiome of the female reproductive system and the impact of the microbial flora composition on IVF outcomes. Studies examining the role of microbiome composition in assisted reproduction suggest that colonization of the transfer catheter tip with Lactobacillus crispatus at the time of embryo transfer may increase implantation and live birth rates.

Conversely, it has been shown that a progesterone-resistant endometrium can result in an altered vaginal microbiome [30].

Last year, a study conducted by Weijue Su et al. compared 16S rRNA gene sequencing and metagenomic analysis of reproductive tracts using QIIME2 and HUMAnN2, investigating the timing of embryo implantation failure in 239 infertile patients and 17 healthy women [47]. This study found a significant correlation between the abundance of lactobacilli and successful embryo implantation (IS) after in vitro fertilization [48]. Functional metagenomic analysis of vaginal samples from infertile patients revealed, compared to healthy controls, increased vaginal pH and a decreased lactobacilli population. These findings once again underscore the correlation between microbiome composition—particularly the abundance of lactobacilli—and fertility or IVF success [47].

In 2018, Koedooder et al. conducted a prospective study involving 300 women of reproductive age who were candidates for in vitro fertilization [49]. Prior to starting the treatment, these women provided midstream urine samples and vaginal swabs. The urinary and vaginal microbiomes were analyzed using both next-generation sequencing (NGS) and IS-pro techniques. The primary endpoint of the study was the achievement of pregnancies after fresh embryo transfer. Based on the microbiome composition, the women were divided into two clusters: pregnant and non-pregnant women. The bacterial species that dominated in predicting pregnancy outcomes were identified and used to create a predictive algorithm. The test panel showed a specificity of 96% and a sensitivity of 81%.

The authors of the study note that the predictive species identified have yet to be confirmed in an independent study. The most significant finding of this predictive test is its high specificity, which suggests that it may be useful in predicting cases where pregnancy is unlikely following treatment. In the future, this could help identify women who should not undergo IVF treatment due to a high probability of not becoming pregnant. To explain the results of the study, it was hypothesized that the microbiome might act as a sensor for the immunological tolerance within the secretory epithelia of individual women [49].

Another hypothesis is that fertility-positive bacterial species may sustain the nutritional environment within epithelial secretions, which could be important for the initial survival of the embryo after transfer to the uterus [50,51].

Koedooder et al.’s study, along with other research papers [52,53], reaffirms the role of *Lactobacillus* in reproductive health and outcomes. Recent studies have shown that infertile women have fewer *Lactobacillus* species compared to fertile women [53,54]. In addition, a microbiome characterized by the prevalence of species other than lactobacilli (such as Gardnerella and Streptococcus) appears to be significantly associated with lower rates of implantation, ongoing pregnancy, and live birth [29,52,55].

A cohort of 31 patients undergoing assisted reproductive therapy (ART) with either their own or donated gametes, followed by the cryotransfer of a single euploid blastocyst, was examined. Two vaginal swabs were collected from each participant immediately prior to embryo transfer [56]. To assess the composition of the vaginal microbiota, the V3–V4 region of the 16S rRNA gene was sequenced, and the data were processed using QIIME2, Bioconductor Phyloseq, and MicrobiomeAnalyst tools. Of the total group, 14 women (45.2%) did not achieve pregnancy, whereas 17 (54.8%) successfully conceived. The vaginal microbiota of women who became pregnant showed a greater prevalence of Lactobacillus species. Cluster analysis identified two main groups: the first group included the genera *Lactobacillus*, *Gardnerella*, *Clostridium*, *Staphylococcus*, and *Dialister*, while the second group consisted of all other genera. In women who achieved pregnancy, microorganisms from the first cluster were predominantly detected [56].

In 2019, Hashimoto et al. studied pregnancy outcomes by comparing women with an endometrium exhibiting a healthy microbial composition (eubiotic) to those with an endometrium characterized by dysbiosis, aiming to identify which microbial composition was most conducive to embryo implantation [57]. The endometrial microbiome of 99 patients under the age of 40 undergoing blastocyst transfer was analyzed. Endometrial samples were collected at the time of transfer, and bacterial profiles were analyzed, focusing on the relative abundance of bacteria, particularly lactobacilli. Thirty-one patients (31.3%) had a dysbiotic endometrium. The background profiles, pregnancy rates per transfer (52.9% vs. 54.8%), and miscarriage rates (11.1% vs. 5.9%) were comparable between patients with eubiotic or dysbiotic endometria. The main bacterial genera found in the dysbiotic endometrium, other than *Lactobacillus*, included *Atopobium*, *Gardnerella*, and *Streptococcus*. In this study, the endometrial bacterial profiles of pregnant women with dysbiotic endometria were similar to those of non-pregnant women [57].

An interesting study investigated the link between the endometrial microbiome and recurrent implantation failure (RIF) [58], given that chronic endometritis is already known to be associated with poor implantation outcomes. Patients with recurrent implantation failure, both with and without chronic endometritis, were enrolled, and cervical and endometrial samples were collected for 16S rRNA gene sequencing. The endometrial microbiota of patients with chronic endometritis showed greater diversity compared to those without endometritis. Linear discriminant analysis (LDA) identified *Proteobacteria*, *Aminicenantales*, and *Chloroflexaceae* as characteristic of chronic endometritis, while the presence of *Lactobacillus*, *Acinetobacter*, *Herbaspirillum*, *Ralstonia*, *Shewanella*, and *Micrococcaceae* was correlated with cases without chronic endometritis. Associations between chronic endometritis, adverse reproductive outcomes, and the predominance of certain bacterial species were also demonstrated in this and other studies [59]. Patients with recurrent implantation failure and chronic endometritis exhibited an endometrial microbial composition associated with poorer reproductive outcomes. Increased microbial diversity and changes in metabolic pathways in patients with chronic endometritis suggest a potential correlation with reproductive outcomes, although further investigation is needed to clarify the causal link between microbiome alterations and fertility [58].

In the future, modulation of the endometrial microbiome and targeted treatments for endometrial dysbiosis could represent new strategies to improve pregnancy outcomes in IVF pathways for patients suffering from chronic endometritis.

As discussed in previous sections, bacterial vaginosis (BV) is a common condition of the female genital tract, with a prevalence of approximately 19% in infertile women. BV is often subclinical and characterized by changes in the vaginal microbial composition, shifting from a predominance of *Lactobacillus* spp. to a more heterogeneous environment with anaerobic bacteria, such as *Gardnerella vaginalis* and *Atopobium vaginae*. Several studies have suggested that BV negatively impacts fecundity.

A prospective study by Haahr et al. highlighted how the vaginal microbiome of patients undergoing IVF can be analyzed using qPCR tests, which may become promising tools in the future for diagnosing vaginal dysbiosis and predicting clinical pregnancy outcomes in IVF treatment [52]. This study involved 130 infertile patients, 90% of whom were Caucasian, who underwent fertility treatment at two different Danish clinics from April to December 2014. Samples were obtained from the posterior vaginal fornix of these women via vaginal swabbing. Gram-stained slides were evaluated according to Nugent’s criteria. PCR primers were used to target four common *Lactobacillus* spp., *Gardnerella vaginalis*, and *Atopobium vaginae*. Threshold levels were established using ROC curve analysis.

The prevalence of bacterial vaginosis, defined by Nugent score [41], was 21% (27/130), while the prevalence of an altered vaginal microbiota, defined by qPCR with high concentrations of *Gardnerella vaginalis* and/or *Atopobium vaginae*, was 28% (36/130). The qPCR diagnostic approach demonstrated a sensitivity and specificity of 93% each for diagnosing BV, as defined by Nugent’s criteria. Of the 84 patients who completed IVF treatment, the overall clinical pregnancy rate was 35% (29/84). This study found that only 9% (2/22) of infertile women with abnormal vaginal microbiota, defined by qPCR, achieved a clinical pregnancy [52].

Therefore, both endometrial microbial composition and vaginal dysbiosis can negatively impact the likelihood of positive clinical pregnancy outcomes in patients undergoing IVF. If the negative correlation between an altered vaginal microbiome and clinical pregnancy rates is definitively confirmed, infertile women suffering from this condition could be screened and treated for dysbiosis before beginning fertility treatments.

A very recent study (March 2025) compared the vaginal microbiome of two types of infertile women undergoing IVF—those with polycystic ovary syndrome (PCOS) and those with tubal factor infertility (TFI)—with normal control women during the implantation period [60]. This study investigated the impact of different vaginal microbiome compositions on fertility treatment outcomes. Vaginal swabs were collected from IVF patients (n = 85) prior to embryo transfer and from normal control women (n = 37) during the 6–8 days following ovulation detection. The results revealed significant differences in the vaginal microbial composition between infertile women with PCOS and TFI undergoing IVF.

The study showed a greater predominance of *Lactobacillus iners* in the non-pregnant group compared to the pregnant group. Additionally, *Pseudomonas* spp. were more abundant in both non-pregnant groups of infertile women [60].

Once again, scientific findings suggest that the composition of the vaginal microbiota, and the female reproductive tract in general, impacts IVF outcomes. The preponderance of *Lactobacillus iners* before embryo transfer may indicate a higher likelihood of IVF failure, while the presence of *Pseudomonas* spp. in the vagina could be an adverse factor for achieving pregnancy.

In 2020, a research group analyzed the vaginal microbiota and metabolomes of patients with unexplained recurrent implantation failure (RIF), while women who achieved clinical pregnancy in the first frozen embryo transfer (FET) cycle were used as controls [61]. The study focused on 16S rRNA gene sequencing of the vaginal microbiome. The results revealed a significant positive correlation between vaginal *Lactobacillus* and pregnancy rate, with patients affected by recurrent implantation failure exhibiting greater microbial diversity than the control group. The metabolomic profile identified 2507 metabolites, of which 37 were significantly different between the two groups. Among these, cyclic 2′,3-UMP and inositol phosphate were found to be the most abundant metabolites in the RIF group, while glycerophospholipids and benzopyran were the lowest metabolites in the RIF group. Benzopyran, as a selective modulator of the estrogen receptor, may influence pregnancy outcomes. These changes in metabolite profiles are likely attributed to the different microbial compositions observed in RIF patients. Furthermore, significant differences in both the vaginal microbiome and metabolomes were demonstrated between patients with unexplained RIF and women who achieved pregnancy in the first FET cycle [61].

In May 2019, another study involving 120 female patients enrolled in IVF treatment was published [62]. Vaginal samples from these patients were sequenced using the V4 region of the 16S ribosomal RNA gene, with genomic clades of *Gardnerella vaginalis* being clustered. The composition of the altered vaginal microbiome was assessed using microscopy and quantitative polymerase chain reaction (qPCR), targeting *Gardnerella vaginalis* and/or *Atopobium vaginae* above defined threshold levels. Three primary microbial community profiles were identified: one dominated by *Lactobacillus crispatus*, another by *Lactobacillus iners*, and a mixed or diverse community type marked by high levels of both *L. crispatus* and *L. iners*, along with commonly shared operational taxonomic units (OTUs). While no statistically significant link was found between these community types and reproductive outcomes, the findings suggested that 16S rRNA gene sequencing did not offer greater predictive accuracy than qPCR. Given its lower cost and practical applicability, qPCR may be a preferable tool for assessing the risk of negative reproductive outcomes in IVF patients [62].

## 4. Discussion

By analyzing and delving into the various studies conducted in recent years to establish an association between the vaginal microbiome and infertility, we have gained insight into how an altered microbial flora, whether in terms of species or their relative abundance, can contribute to the onset of subfertility or infertility in women.

A healthy genital microbial flora is predominantly composed of lactobacilli, which, through the production of lactic acid, create an unfavorable environment for the growth of pathogenic bacteria and reduce the likelihood of infections. Additionally, a genital tract rich in lactobacilli is associated with a lower risk of miscarriage and preterm birth. Lactobacilli also play a key role in creating a favorable environment for embryo implantation by producing lactic acid, hydrogen peroxide, bacteriocins, hydroxyl radicals, and probiotics.

Bacterial vaginosis (BV), one of the most common conditions affecting the female genital system, is characterized by an altered microbial flora that results in an increase in *Gardnerella vaginalis* at the expense of *Lactobacilli*. BV is associated with endometritis, infertility, and pelvic inflammatory disease. It has been found that approximately 40 percent of infertile patients undergoing IVF have an altered genital microbiome.

Modern techniques, such as next-generation sequencing (NGS) [63] and 16S RNA sequencing, have been pivotal in advancing our understanding of the microbiome and its relationship to infertility. These technologies have provided valuable insights into how the microbiome influences reproductive health [64,65].

Various approaches have been used to connect the microbiome with IVF techniques. For instance, the tip of the embryo transfer catheter has been analyzed from a bacteriological perspective. Attention has also been given to the correlation between the presence of lactobacilli and embryo implantation success rates. Not only vaginal and cervical samples, but also urine samples, have been processed to better characterize the female genitourinary environment.

It is clear that a higher proportion of lactobacilli in the vaginal microbiome is a positive factor for the potential success of IVF, particularly embryo transfer. Ensuring the treatment of any dysbiosis and optimizing the vaginal, cervical, and endometrial environment should be a primary goal when preparing women for IVF treatments.

Deepening the study of the composition of vaginal microbial flora in women undergoing therapeutic IVF pathways could have significant implications in clinical practice in the near future. Cases of couple infertility often classified as idiopathic could be used to find a diagnostic definition through confirmation of a microbial alteration of the genital tract. In addition, it would be useful in connection with the study of the genital microbiome to formulate personalized treatment protocols in the case of dysbiosis or frankly pathological situations in order to promote not only conception and embryo implantation but also a smooth course of pregnancy. It might be useful to systematically study the microbiome of women, particularly those with idiopathic infertility, repeated miscarriages, and numerous failures of medically assisted procreation techniques, in order to provide a diagnostic answer to the problem of many couples and improve outcomes following embryo transfer.

Looking ahead, it may be possible to develop algorithms that, through accurate analysis of the microbial composition of both the upper and lower genital tracts, could predict the success or failure of IVF techniques. Our hope is that science will continue to advance in further elucidating the relationship between the female genital microbiome, fertility, and the outcomes of assisted reproductive technologies.

We hope that in the future, with the help of new technologies and artificial intelligence [63,66,67,68,69], it will be possible to systematically analyze the genital microbiome of a woman undergoing IVF treatment in order to identify and treat those factors that may hinder the achievement of pregnancy.

## 5. Limitations

One of the limitations of this review concerns its exclusive focus on the female microbiome, which led to the exclusion of literature related to the male microbiome, despite it being briefly mentioned in the Introduction. This choice was guided by the specific objective of the study, as outlined in the inclusion and exclusion criteria; however, it still represents a methodological limitation, as we acknowledge that it may not provide a complete and bilateral view of microbiotic interactions within infertile couples.

It should be noted, however, that based on a brief search of the databases used, scientific literature on the male microbiome remains underrepresented and less developed compared to that on the female counterpart. The available studies are limited both in number and methodological quality, making it difficult, in our opinion, to draw solid conclusions about the impact of the male microbiome on fertility and the outcomes of assisted reproductive technologies.

This gap highlights the need for future research that includes both partners, within a more integrated and systemic perspective of couple fertility—an aspect we will certainly aim to explore in future studies.

## Data Availability

The raw data supporting the conclusions of this article will be made available by the authors upon request.

## References

[1] O’Hara A.M., Shanahan F. (2006). The Gut Flora as a Forgotten Organ. EMBO Rep..

[2] Venneri M.A., Franceschini E., Sciarra F., Rosato E., D’Ettorre G., Lenzi A. (2022). Human Genital Tracts Microbiota: Dysbiosis Crucial for Infertility. J. Endocrinol. Investig..

[3] Magill R.G., MacDonald S.M. (2023). Male Infertility and the Human Microbiome. Front. Reprod. Health.

[4] Infertility. https://www.who.int/news-room/fact-sheets/detail/infertility.

[5] Alimena S., Davis J., Fichorova R.N., Feldman S. (2022). The Vaginal Microbiome: A Complex Milieu Affecting Risk of Human Papillomavirus Persistence and Cervical Cancer. Curr. Probl. Cancer.

[6] Balla B., Illés A., Tobiás B., Pikó H., Beke A., Sipos M., Lakatos P., Kósa J.P. (2024). The Role of the Vaginal and Endometrial Microbiomes in Infertility and Their Impact on Pregnancy Outcomes in Light of Recent Literature. Int. J. Mol. Sci..

[7] Gullo G., Basile G., Cucinella G., Greco M.E., Perino A., Chiantera V., Marinelli S. (2023). Fresh vs. Frozen Embryo Transfer in Assisted Reproductive Techniques: A Single Center Retrospective Cohort Study and Ethical-Legal Implications. Eur. Rev. Med. Pharmacol. Sci..

[8] Sciorio R., De Paola L., Notari T., Ganduscio S., Amato P., Crifasi L., Marotto D., Billone V., Cucinella G., Perino A. (2025). Decoding the Puzzle of Male Infertility: The Role of Infection, Inflammation, and Autoimmunity. Diagnostics.

[9] Cucinella G., Gullo G., Catania E., Perino A., Billone V., Marinelli S., Napoletano G., Zaami S. (2024). Stem Cells and Infertility: A Review of Clinical Applications and Legal Frameworks. J. Pers. Med..

[10] Xiao L., Zuo Z., Zhao F. (2024). Microbiome in Female Reproductive Health: Implications for Fertility and Assisted Reproductive Technologies. Genom. Proteom. Bioinform..

[11] Claudiac Recent Insights into the Vaginal Microbiota. https://www.microbiotajournal.com/article/771.

[12] Gullo G., Scaglione M., Laganà A.S., Perino A., Andrisani A., Chiantera V., Cucinella G., Gitas G., Barra F., Riemma G. (2023). Assisted Reproductive Techniques and Risk of Congenital Heart Diseases in Children: A Systematic Review and Meta-Analysis. Reprod. Sci..

[13] Gullo G., Scaglione M., Cucinella G., Perino A., Chiantera V., D’Anna R., Laganà A.S., Buzzaccarini G. (2022). Impact of Assisted Reproduction Techniques on the Neuro-Psycho-Motor Outcome of Newborns: A Critical Appraisal. J. Obstet. Gynaecol..

[14] Vergallo G.M., Marinelli S., Napoletano G., De Paola L., Treglia M., Zaami S., Frati P. (2025). 20 Years Since the Enactment of Italian Law No. 40/2004 on Medically Assisted Procreation: How It Has Changed and How It Could Change. Int. J. Environ. Res. Public Health.

[15] Smeenk J., Wyns C., De Geyter C., Kupka M., Bergh C., Cuevas Saiz I., De Neubourg D., Rezabek K., Tandler-Schneider A., European IVF Monitoring Consortium (EIM) for the European Society of Human Reproduction and Embryology (ESHRE) (2023). ART in Europe, 2019: Results Generated from European Registries by ESHRE. Hum. Reprod..

[16] Tester R., Al-Ghazzewi F.H. (2018). Intrinsic and Extrinsic Carbohydrates in the Vagina: A Short Review on Vaginal Glycogen. Int. J. Biol. Macromol..

[17] France M., Alizadeh M., Brown S., Ma B., Ravel J. (2022). Towards a Deeper Understanding of the Vaginal Microbiota. Nat. Microbiol..

[18] Günther V., Allahqoli L., Watrowski R., Maass N., Ackermann J., von Otte S., Alkatout I. (2022). Vaginal Microbiome in Reproductive Medicine. Diagnostics.

[19] Freitas A.C., Bocking A., Hill J.E., Money D.M., VOGUE Research Group (2018). Increased Richness and Diversity of the Vaginal Microbiota and Spontaneous Preterm Birth. Microbiome.

[20] Delgado-Diaz D.J., Tyssen D., Hayward J.A., Gugasyan R., Hearps A.C., Tachedjian G. (2019). Distinct Immune Responses Elicited from Cervicovaginal Epithelial Cells by Lactic Acid and Short Chain Fatty Acids Associated with Optimal and Non-Optimal Vaginal Microbiota. Front. Cell Infect. Microbiol..

[21] Punzón-Jiménez P., Labarta E. (2021). The Impact of the Female Genital Tract Microbiome in Women Health and Reproduction: A Review. J. Assist. Reprod. Genet..

[22] Wee B.A., Thomas M., Sweeney E.L., Frentiu F.D., Samios M., Ravel J., Gajer P., Myers G., Timms P., Allan J.A. (2018). A Retrospective Pilot Study to Determine Whether the Reproductive Tract Microbiota Differs between Women with a History of Infertility and Fertile Women. Aust. N. Z. J. Obstet. Gynaecol..

[23] Chen C., Song X., Wei W., Zhong H., Dai J., Lan Z., Li F., Yu X., Feng Q., Wang Z. (2017). The Microbiota Continuum along the Female Reproductive Tract and Its Relation to Uterine-Related Diseases. Nat. Commun..

[24] Pelzer E.S., Willner D., Buttini M., Huygens F. (2018). A Role for the Endometrial Microbiome in Dysfunctional Menstrual Bleeding. Antonie Van Leeuwenhoek.

[25] Winters A.D., Romero R., Gervasi M.T., Gomez-Lopez N., Tran M.R., Garcia-Flores V., Pacora P., Jung E., Hassan S.S., Hsu C.-D. (2019). Does the Endometrial Cavity Have a Molecular Microbial Signature?. Sci. Rep..

[26] Baker J.M., Chase D.M., Herbst-Kralovetz M.M. (2018). Uterine Microbiota: Residents, Tourists, or Invaders?. Front. Immunol..

[27] Toson B., Simon C., Moreno I. (2022). The Endometrial Microbiome and Its Impact on Human Conception. Int. J. Mol. Sci..

[28] Mitchell C.M., Haick A., Nkwopara E., Garcia R., Rendi M., Agnew K., Fredricks D.N., Eschenbach D. (2015). Colonization of the Upper Genital Tract by Vaginal Bacterial Species in Nonpregnant Women. Am. J. Obstet. Gynecol..

[29] Moreno I., Codoñer F.M., Vilella F., Valbuena D., Martinez-Blanch J.F., Jimenez-Almazán J., Alonso R., Alamá P., Remohí J., Pellicer A. (2016). Evidence That the Endometrial Microbiota Has an Effect on Implantation Success or Failure. Am. J. Obstet. Gynecol..

[30] Sirota I., Zarek S.M., Segars J.H. (2014). Potential Influence of the Microbiome on Infertility and Assisted Reproductive Technology. Semin. Reprod. Med..

[31] Hawes S.E., Hillier S.L., Benedetti J., Stevens C.E., Koutsky L.A., Wolner-Hanssen P., Holmes K.K. (1996). Hydrogen Peroxide-Producing Lactobacilli and Acquisition of Vaginal Infections. J. Infect. Dis..

[32] Aroutcheva A.A., Simoes J.A., Behbakht K., Faro S. (2001). Gardnerella Vaginalis Isolated from Patients with Bacterial Vaginosis and from Patients with Healthy Vaginal Ecosystems. Clin. Infect. Dis..

[33] Ng S.C., Hart A.L., Kamm M.A., Stagg A.J., Knight S.C. (2009). Mechanisms of Action of Probiotics: Recent Advances. Inflamm. Bowel Dis..

[34] Moreno I., Garcia-Grau I., Perez-Villaroya D., Gonzalez-Monfort M., Bahçeci M., Barrionuevo M.J., Taguchi S., Puente E., Dimattina M., Lim M.W. (2022). Endometrial Microbiota Composition Is Associated with Reproductive Outcome in Infertile Patients. Microbiome.

[35] Spiegel C.A., Amsel R., Eschenbach D., Schoenknecht F., Holmes K.K. (1980). Anaerobic Bacteria in Nonspecific Vaginitis. N. Engl. J. Med..

[36] Leitich H., Kiss H. (2007). Asymptomatic Bacterial Vaginosis and Intermediate Flora as Risk Factors for Adverse Pregnancy Outcome. Best. Pract. Res. Clin. Obstet. Gynaecol..

[37] Leitich H., Bodner-Adler B., Brunbauer M., Kaider A., Egarter C., Husslein P. (2003). Bacterial Vaginosis as a Risk Factor for Preterm Delivery: A Meta-Analysis. Am. J. Obstet. Gynecol..

[38] Paladine H.L., Desai U.A. (2018). Vaginitis: Diagnosis and Treatment. Am. Fam. Physician.

[39] Spiegel C.A., Amsel R., Holmes K.K. (1983). Diagnosis of Bacterial Vaginosis by Direct Gram Stain of Vaginal Fluid. J. Clin. Microbiol..

[40] Amsel R., Totten P.A., Spiegel C.A., Chen K.C., Eschenbach D., Holmes K.K. (1983). Nonspecific Vaginitis. Diagnostic Criteria and Microbial and Epidemiologic Associations. Am. J. Med..

[41] Challa A., Sood S., Kachhawa G., Upadhyay A.D., Dwivedi S.N., Gupta S. (2022). Diagnostic Concordance between Amsel’s Criteria and the Nugent Scoring Method in the Assessment of Bacterial Vaginosis. Sex. Health.

[42] Borges S., Silva J., Teixeira P. (2014). The Role of Lactobacilli and Probiotics in Maintaining Vaginal Health. Arch. Gynecol. Obstet..

[43] Shipitsyna E., Roos A., Datcu R., Hallén A., Fredlund H., Jensen J., Engstrand L., Unemo M. (2013). Composition of the Vaginal Microbiota in Women of Reproductive Age—Sensitive and Specific Molecular Diagnosis of Bacterial Vaginosis Is Possible?. PLoS ONE.

[44] Swidsinski A., Loening-Baucke V., Mendling W., Dörffel Y., Schilling J., Halwani Z., Jiang X., Verstraelen H., Swidsinski S. (2014). Infection through Structured Polymicrobial Gardnerella Biofilms (StPM-GB). Histol. Histopathol..

[45] García-Velasco J.A., Menabrito M., Catalán I.B. (2017). What Fertility Specialists Should Know about the Vaginal Microbiome: A Review. Reprod. Biomed. Online.

[46] Machado A., Cerca N. (2015). Influence of Biofilm Formation by Gardnerella Vaginalis and Other Anaerobes on Bacterial Vaginosis. J. Infect. Dis..

[47] Su W., Gong C., Zhong H., Yang H., Chen Y., Wu X., Jin J., Xi H., Zhao J. (2024). Vaginal and Endometrial Microbiome Dysbiosis Associated with Adverse Embryo Transfer Outcomes. Reprod. Biol. Endocrinol..

[48] Zaami S. (2018). Assisted Heterologous Fertilization and the Right of Donorconceived Children to Know Their Biological Origins. Clin. Ter..

[49] Koedooder R., Singer M., Schoenmakers S., Savelkoul P.H.M., Morré S.A., de Jonge J.D., Poort L., Cuypers W.-J.S.S., Budding A.E., Laven J.S.E. (2018). The ReceptIVFity Cohort Study Protocol to Validate the Urogenital Microbiome as Predictor for IVF or IVF/ICSI Outcome. Reprod. Health.

[50] Brosens J.J., Salker M.S., Teklenburg G., Nautiyal J., Salter S., Lucas E.S., Steel J.H., Christian M., Chan Y.-W., Boomsma C.M. (2014). Uterine Selection of Human Embryos at Implantation. Sci. Rep..

[51] Templeton A., Morris J.K., Parslow W. (1996). Factors That Affect Outcome of In-Vitro Fertilisation Treatment. Lancet.

[52] Haahr T., Jensen J.S., Thomsen L., Duus L., Rygaard K., Humaidan P. (2016). Abnormal Vaginal Microbiota May Be Associated with Poor Reproductive Outcomes: A Prospective Study in IVF Patients. Hum. Reprod..

[53] Graspeuntner S., Bohlmann M.K., Gillmann K., Speer R., Kuenzel S., Mark H., Hoellen F., Lettau R., Griesinger G., König I.R. (2018). Microbiota-Based Analysis Reveals Specific Bacterial Traits and a Novel Strategy for the Diagnosis of Infectious Infertility. PLoS ONE.

[54] Kyono K., Hashimoto T., Nagai Y., Sakuraba Y. (2018). Analysis of Endometrial Microbiota by 16S Ribosomal RNA Gene Sequencing among Infertile Patients: A Single-Center Pilot Study. Reprod. Med. Biol..

[55] Mangot-Bertrand J., Fenollar F., Bretelle F., Gamerre M., Raoult D., Courbiere B. (2013). Molecular Diagnosis of Bacterial Vaginosis: Impact on IVF Outcome. Eur. J. Clin. Microbiol. Infect. Dis..

[56] Bernabeu A., Lledo B., Díaz M.C., Lozano F.M., Ruiz V., Fuentes A., Lopez-Pineda A., Moliner B., Castillo J.C., Ortiz J.A. (2019). Effect of the Vaginal Microbiome on the Pregnancy Rate in Women Receiving Assisted Reproductive Treatment. J. Assist. Reprod. Genet..

[57] Hashimoto T., Kyono K. (2019). Does Dysbiotic Endometrium Affect Blastocyst Implantation in IVF Patients?. J. Assist. Reprod. Genet..

[58] Zhang H., Zou H., Zhang C., Zhang S. (2024). Chronic Endometritis and the Endometrial Microbiota: Implications for Reproductive Success in Patients with Recurrent Implantation Failure. Ann. Clin. Microbiol. Antimicrob..

[59] Gullo G., Scaglione M., Cucinella G., Riva A., Coldebella D., Cavaliere A.F., Signore F., Buzzaccarini G., Spagnol G., Laganà A.S. (2022). Congenital Zika Syndrome: Genetic Avenues for Diagnosis and Therapy, Possible Management and Long-Term Outcomes. J. Clin. Med..

[60] Zhao H., Wang C., Narsing Rao M.P., Rafiq M., Luo G., Li S., Kang Y.-Q. (2025). Effects of Vaginal Microbiota on in Vitro Fertilization Outcomes in Women with Different Infertility Causes. Microbiol. Spectr..

[61] Fu M., Zhang X., Liang Y., Lin S., Qian W., Fan S. (2020). Alterations in Vaginal Microbiota and Associated Metabolome in Women with Recurrent Implantation Failure. mBio.

[62] Haahr T., Humaidan P., Elbaek H.O., Alsbjerg B., Laursen R.J., Rygaard K., Johannesen T.B., Andersen P.S., Ng K.L., Jensen J.S. (2019). Vaginal Microbiota and In Vitro Fertilization Outcomes: Development of a Simple Diagnostic Tool to Predict Patients at Risk of a Poor Reproductive Outcome. J. Infect. Dis..

[63] Wensel C.R., Pluznick J.L., Salzberg S.L., Sears C.L. (2022). Next-Generation Sequencing: Insights to Advance Clinical Investigations of the Microbiome. J. Clin. Investig..

[64] Davies R., Minhas S., Jayasena C.N. (2023). Next-Generation Sequencing to Elucidate the Semen Microbiome in Male Reproductive Disorders. Medicina.

[65] Doroftei B., Ilie O.-D., Anton N., Armeanu T., Ilea C. (2022). A Mini-Review Regarding the Clinical Outcomes of In Vitro Fertilization (IVF) Following Pre-Implantation Genetic Testing (PGT)-Next Generation Sequencing (NGS) Approach. Diagnostics.

[66] Miloski B. (2023). Opportunities for Artificial Intelligence in Healthcare and in Vitro Fertilization. Fertil. Steril..

[67] Cheng X., Joe B. (2023). Artificial Intelligence in Medicine: Microbiome-Based Machine Learning for Phenotypic Classification. Methods Mol. Biol..

[68] Marinelli S., De Paola L., Stark M., Montanari Vergallo G. (2025). Artificial Intelligence in the Service of Medicine: Current Solutions and Future Perspectives, Opportunities, and Challenges. Clin. Ter..

[69] Jiang V.S., Bormann C.L. (2023). Artificial Intelligence in the in Vitro Fertilization Laboratory: A Review of Advancements over the Last Decade. Fertil. Steril..

